# Single incision endoscopic strip craniectomy for sagittal craniosynostosis

**DOI:** 10.3171/2021.1.FOCVID20120

**Published:** 2021-04-01

**Authors:** Edward S. Ahn, Archis R. Bhandarkar

**Affiliations:** 1 Department of Neurologic Surgery, Mayo Clinic, Rochester; and; 2 Mayo Clinic Alix School of Medicine, Rochester, Minnesota

**Keywords:** craniosynostosis, endoscopic, ultrasonic bone cutting

## Abstract

The authors describe an endoscopic strip craniectomy through a single incision for the treatment of sagittal craniosynostosis in a young infant. The endoscopic strip craniectomy was first introduced with the use of two incisions on either end of the fused suture. This single-incision technique offers several advantages. There is a cosmetic advantage and a reduced risk of wound complications. This technique also allows for early control of emissary veins and an inside-out identification of the lambdoid sutures. Endoscopic visualization is optimized to reduce the risk of blood loss, especially because circulating blood volume is very limited in these young infants.

The video can be found here: https://vimeo.com/514366415

## Transcript

**0:23** We are presenting single-incision endoscopic strip craniectomy for the treatment of sagittal craniosynostosis.

**0:33** The case is a standard presentation of a young infant who is 5 weeks old with scaphocephaly and a palpable midline ridge at the vertex. The physical examination is consistent with sagittal craniosynostosis. The measured cephalic index is 66. No imaging studies were performed. The baby's weight is 4.6 kg. We recommended endoscopic surgery followed by helmeting.

**1:01** For this procedure, the baby was positioned supine with her head turned toward the surgeon.^1^ We could palpate the anterior fontanelle and made a transverse incision posterior to it. Then, under direct visualization, we made a 3-cm-wide craniectomy that started at the posterior border of the fontanelle. This anterior location for the approach was chosen over a posterior prelambdoid one because of the relative thinness and avascularity of the bone in this region. As a result, we could start the craniectomy with rongeurs and eliminated the need for drilling a burr hole. We then proceeded to the endoscopic strip craniectomy.^2^

**1:41** We used a 30° Storz endoscope. Attached to it was a Storz optical dissector which was used for retraction of the tissues.

**1:51** We used a combination of the dissector and a periosteal elevator to dissect the scalp from the pericranium. With this maneuver, we exposed the fused sagittal suture toward the vertex. Then we cauterized the pericranium where we planned to make our parasagittal cuts in the bone.

**2:10** Then we rotated the dissector to begin the epidural dissection underneath the bone. We used the dissector to retract the underlying dura with the aim of identifying any emissary veins associated with the sagittal sinus. We used the bipolar to cauterize any dural attachments or visible veins.

**2:32** The fused sagittal suture is easily visible above. We followed this suture all the way until we felt the resistance of the dural attachment at the lambdoid sutures, thus completing the epidural dissection.

**2:42** Then with retraction of the dura and scalp, we made parasagittal cuts with an ultrasonic bone cutter. We prefer this device for its hemostatic properties, but Tessier bone scissors would also suffice. Then we removed the midline bone in piecemeal fashion with rongeurs.

**3:04** It is important to note that bleeding from emissary veins may occur during this process and should be controlled immediately.

**3:19** Then we rotated the retractor back for the last portion of the craniectomy.

**3:25** We could dissect and identify this last rim of bone with its dural attachment at the lambdoid sutures. We used a rongeur to morselize the bone and remove it piecemeal from the lambdoid sutures.

**3:51** This patient had a fairly sloped vertex downward, so we used an angled rongeur to remove the bone that was over the slope. Then we confirmed that we had completely exposed the medial lambdoid sutures and there were no remaining pieces of bone.

**4:07** One final view of the midline dura confirmed hemostasis and no jagged edges on the craniectomy. At the end, the medial lambdoid sutures were exposed and the craniectomy was complete.

**4:30** This patient did not require a blood transfusion. She was discharged home on postoperative day 1. She began wearing the cranial molding orthosis on postoperative day 11.

**4:41** Follow-up at 1 and 3 months after surgery showed correction of the cephalic index to 76 and 80, respectively. There was good healing of the single incision behind the fontanelle.

**4:58** The first author has used this single-incision approach without complication in all patients who underwent endoscopic strip craniectomy for sagittal craniosynostosis over a 5-year period. In this consecutive series of 41 patients, the mean age at surgery was 13.6 weeks. The weight was 6 kg. The length of stay was 1.2 days. The final cephalic index at last follow-up was 78.1. There were three blood transfusions. No patients underwent revision surgery for the craniosynostosis.

**5:35** In summary, we believe there are several advantages to this technique that eliminates one incision from the traditional two-incision approach.^3–5^ There is a cosmetic advantage. In addition, there is a decreased risk of wound dehiscence or infection that is not uncommonly seen, particularly with pressure from the helmet. We have found that the lambdoid suture is easily identified from this inside-out approach as opposed to the use of external landmarks or palpation. This inside-out approach also allows for control of emissary veins with endoscopic visualization. This technique also eliminates the need for burr holes. We think these advantages help with early identification of bleeding sources and thereby reduce the risk of blood loss in these cases where hemostasis is critical.

## Author Contributions

Primary surgeon: Ahn. Editing and drafting the video and abstract: both authors. Critically revising the work: both authors. Reviewed submitted version of the work: both authors. Approved the final version of the work on behalf of both authors: Ahn. Supervision: Ahn.
